# Mechanical Characteristics of Tara Gum/Orange Peel Films Influenced by the Synergistic Effect on the Rheological Properties of the Film-Forming Solutions

**DOI:** 10.3390/polym17131767

**Published:** 2025-06-26

**Authors:** Nedelka Juana Ortiz Cabrera, Luis Felipe Miranda Zanardi, Martin Alberto Massuelli

**Affiliations:** 1 Departamento de Ingeniería Química, Universidad Nacional de San Agustín, Arequipa 04000, Peru; nortizc@unsa.edu.pe; 2 INFAP-CONICET-UNSL, Departamento de Química, Facultad de Química, Bioquimica y Farmacia, Universidad Nacional de San Luis, San Luis, Ejercito de los Andes 950, San Luis 5700, Argentina; masuelli@unsl.edu.ar

**Keywords:** film-forming solution, Tara gum, orange peel, rheological properties, mechanical properties

## Abstract

Film-forming solutions were prepared using Tara gum (TG), with glycerol (GL) as a plasticizer and orange peel powder (OP) as a filler. A TG stock solution (10 g/L) was initially prepared to facilitate homogenization, from which appropriate dilutions were made to obtain final concentrations of 0.6%, 0.8%, and 1.0% (*w/v*). GL (30% and 50%) and OP (0%, 20%, and 50%) were incorporated based on the dry weight of TG, meaning their amounts were calculated relative to TG content to ensure consistent formulation ratios. Rheological parameters, including the flow behavior index, consistency coefficient, storage modulus (G′), and loss modulus (G″), were characterized via steady shear and oscillatory rheometry. Mechanical properties, such as the Young’s modulus, tensile strength, and elongation at break, were also evaluated. A strong positive correlation (R^2^ = 0.840) was observed between G′ and the Young’s modulus, indicating that solutions with higher internal network strength yield films with greater stiffness. The synergistic interaction between TG and OP was critical: TG primarily enhanced stiffness and mechanical reinforcement, whereas OP improved structural cohesion and stability. GL functioned as a plasticizer, increasing film flexibility while reducing stiffness. These interactions led to a reduction in film solubility by up to 62.43%, particularly in formulations without orange peel powder. In contrast, mechanical strength increased by up to 50.21% in films containing orange peel powder, as those without it exhibited significantly lower tensile strength. Flexibility, expressed as elongation at break, was enhanced by up to 78.86% in formulations with higher glycerol content. Barrier properties were also improved, demonstrated by decreased water vapor permeability and increased hydrophobicity, attributed to the TG–OP synergy. A regression model (R^2^ = 0.928) substantiated the contributions of TG to stiffness, OP to matrix reinforcement, and GL to flexibility modulation. This study underscores the pivotal role of rheological behavior in defining film performance and presents a novel analytical framework applicable to the design of sustainable, high-performance biopolymeric materials.

## 1. Introduction

Plastic pollution causes significant environmental damage due to its persistence and high greenhouse gas emissions during degradation, contributing to climate change and global warming. This has driven the search for sustainable alternatives, such as bioplastics, which aim to replace conventional plastics with biodegradable and environmentally friendly materials. Among these, hydrocolloid-based films have gained attention due to their ability to form biopolymeric networks with promising mechanical and barrier properties. However, the influence of the rheological behavior of film-forming solutions on the final functional properties of biopolymer films remains an underexplored area.

Tara gum (TG) is a galactomannan widely used as a thickening agent in the food industry and has been successfully applied in edible coatings [1]. Due to its high hydroxyl content, it exhibits a hydrophilic nature, forming hydrogen bonds with water molecules and generating a three-dimensional network with high viscosities at low concentrations [2]. Glycerol (GL) acts as a plasticizer, reducing the intermolecular forces between polymer chains and increasing their flexibility [3]. Meanwhile, orange peel (OP) has been studied as a filler in biopolymeric matrices due to its high cellulose and pectin content, which provide structural reinforcement and bioactive properties [4,5].

Hydrocolloid-based solutions typically exhibit viscoelastic behavior and pseudoplastic flow, where viscosity decreases under shear stress [6,7]. Although these rheological characteristics are well-documented, there is limited research on how they correlate with the mechanical and barrier properties of the resulting biopolymeric films. Recent studies suggest that molecular interactions between hydrocolloids, plasticizers, and fillers can alter the microstructure of the films, affecting their elasticity, tensile strength, water resistance, and overall functional performance [8]. However, the combined effects of TG, GL, and OP on the rheological behavior of film-forming solutions—and how this behavior translates into mechanical reinforcement and barrier optimization—have not been fully explored. This gap in the literature highlights the need to understand the structure–function relationship in such complex biopolymer systems.

This study evaluates the rheological properties of mixtures of TG/GL/OP and their correlation with the mechanical performance of the resulting films. Characterizations such as FTIR and barrier property tests (solubility, permeability, contact angle) support the analysis of component interactions within the polymer matrix. The novelty lies in linking rheological behavior with mechanical properties as a perspective not previously explored.

## 2. Materials and Methods

### 2.1. Materials

The materials used in this research were Tara gum (TG) (Alnicolsa, S.A., Lima, Peru), glycerol (GL) 99.5% pure (Delta Química S.R.L., Arequipa, Peru), and orange peel powder (OP) with a particle size of 160 μm. Orange peels obtained from the steam distillation process for essential oil extraction were received, dried for 4 h at 50 °C, ground using a ball mill, and sieved with ASTM-standard sieves until reaching the desired particle size.

### 2.2. Preparation of Film-Forming Solutions and Film Casting

The film-forming solutions were prepared using Tara gum (TG), with glycerol (GL) as a plasticizer and orange peel powder (OP) as a filler. A TG stock solution (10 g/L) was first prepared to facilitate homogenization and refrigerated for 24 h at 5 °C. From this stock, final concentrations of 0.6%, 0.8%, and 1.0% (*w*/*v*) TG were obtained by dilution. GL was added at concentrations of 30% and 50%, and OP at 0%, 20%, and 50%, both calculated based on the dry weight of TG.

In the first experimental stage, the rheological behavior of TG/GL solutions was evaluated. The diluted TG solutions were mixed with GL (30% and 50%) and stirred at 3000 RPM for 15 min at 80 °C. In the second stage, the effect of OP was assessed by incorporating it into the TG/GL mixtures following the same rheological protocol. Finally, in the third stage, films were prepared by casting 500 mL of the final solutions onto glass plates, followed by drying in a tray dryer at 37 °C for 12 h.

### 2.3. Rheological Measurements

Rheological measurements were performed using an MCR 702 Twindrive Anton Paar rheometer provided with a concentric cylinder geometry following the ISO 3219-2:2021 standard [9]. The analyses were carried out in triplicate at a temperature of 25 °C. The flow curve data were fitted using the Herschel–Bulkley model [10]:

(1) $$\tau \;=\;\tau_{0}\;+\;k\;{\dot{\gamma }}^{n},$$

where τ_0_ is the yield stress (Pa), k is the consistency coefficient (Pa s), n is the flow behavior index, and $\dot{\gamma }$ is the shear rate (s^−1^).

Oscillatory strain sweeps were performed between 0.1 and 400% strain at a frequency of 1 Hz to establish the linear viscoelastic region (LVR). Then, frequency sweeps were performed at a fixed strain within the linear viscoelastic range (5%) to obtain the mechanical spectra: storage modulus, (G′), loss modulus, (G″), and complex viscosity (η*). Dynamic tests were performed at 25 °C and fitted to the Generalized Maxwell Model for G′ and G″ (Equations (2) and (3)) [11]:

(2) $$G\prime \;(\omega )=\sum_{n}\frac{G_{i}\lambda_{i}^{2}\omega^{2}}{\left(1+\lambda_{i}^{2}\omega^{2}\right)},$$

(3) $$G\prime \prime \;(\omega )=\sum_{n}\frac{G_{i}\lambda_{i}\omega }{\left(1+\lambda_{i}^{2}\omega^{2}\right)}$$

where G′ is the storage modulus (Pa), G″ is the loss modulus (Pa), ω is the angular frequency (rad/s), and both G_i_ and λ_i_ correspond to constants and parameters, which are determined from the frequency intervals included in the spectrum.

### 2.4. Film Characterization

#### 2.4.1. Film Thickness

Film thickness was measured with a Mitutoyo 0-1 “electronic digital micrometer having a 0.0005”/0.001 mm resolution. Five random measurements were taken from the biofilms already cut, and the values obtained were averaged.

#### 2.4.2. Water Solubility (%S)

Pieces of 2 cm × 2 cm of the sample films were cut and dried at 100 °C for 24 h to obtain a constant weight, after which the samples were immersed in 100 mL of distilled water for 24 h. Finally, the samples were removed from the water and dried at 100 °C for 24 h. The water solubility of the films was reported as the weight loss of the films, following Equation (4) [12]:

(%S) = W_0_ − W_1_/W_0_ × 100%, (4)

where W_0_ is the initial weight, and W_1_ is the final weight of the films.

#### 2.4.3. Water Vapor Permeability (WVP)

Water vapor permeability (WVP) and water vapor transmission rate (WVTR) were determined in accordance with ASTM E96/E96M-05. The films were cut into 3 cm × 3 cm squares after balancing their relative humidity. The square pieces were sealed in test tubes containing 10 mL of distilled water and placed in a desiccator containing silica gel. The weight of the tubes was checked every hour for 8 h. The slope of each line was calculated by the linear regression (R^2^ > 0.99) of weight change versus time, and WVTR and WVP were calculated based on Equations (5) and (6) [12,13]:

WVTR = Slope/Film area,(5)

WVP = WVTR × X/(PA_1_ − PA_2_), (6)

where X is the film thickness, PA_1_ is the vapor partial pressure on the outer surface of the film in the dryer, and PA_2_ is the vapor partial pressure on the inner surface of the film in the test tube.

#### 2.4.4. Fourier Transform Infrared (FTIR) Spectroscopy

The FTIR spectra of the films were determined using a Spectrum II Perkin Elmer Infrared Spectrophotometer provided with an ATR accessory. Dried film samples of 2 cm × 2 cm were cut and scanned in the range of 4000 cm^−1^ to 450 cm^−1^ wave numbers with 30 accumulations.

#### 2.4.5. Contact Angle (CA)

The contact angle was determined on films following the sessile drop method. A total of 10 μL of distilled water was carefully poured over the surface of the films (2 cm × 2 cm) through a 10 μL micropipette, and the angles were measured before swelling began. Measurements were performed at 25 °C. The reverse and right contact angles were automatically calculated using the software One Attention that controls the Biolin Scientific Optical Tensiometer model Theta T200.

### 2.5. Mechanical Properties of Films

The Multitest 0.5i Mecmesin dynamometer was used to measure the mechanical properties according to ASTM D882-18 with some modifications. The films were first cut into 20 mm × 120 mm specimens and then conditioned at 53 ± 1% relative humidity and 25 ± 1 °C for 72 h before mechanical testing. The initial jaw spacing and crosshead speed used were set at 50 mm and 0.5 mm/s, respectively. The tensile strength (TS) [14], percent elongation (%E) [14], and Young’s modulus (YM) [15] were calculated from the stress vs. strain plot using the following equations:

TS = F/(A × X),(7)

%E = (L − L_1_)/L_1_ × 100%, (8)

YM = TS/((L − L_1_)/L_1_), (9)

where F is the tensile force (N), A is the cross-sectional area (mm^2^), X is the thickness (mm), L_1_ is the initial film length, and L is the film length at the breaking point (mm).

## 3. Results and Discussion

### 3.1. Analysis of the Film-Forming Solutions

The solutions containing lower concentrations of TG were less viscous, and the thickness of the corresponding films were thinner. The total mass of nonwater components in the films (TG, GL, and OP) determines their thicknesses.

Plasticizer addition was carried out at concentrations of 30% and 50%, determined on the basis of preliminary tests. During these tests, plasticizer concentrations ranging from 10% to 100% (on a dry hydrocolloid basis) were evaluated. The results indicated that films obtained with levels below 30% were brittle and difficult to detach, while concentrations above 50% GL saturated the polymeric matrix, preventing proper film formation due to the accumulation of plasticizer on the surface. Therefore, the suitable plasticizer concentration ranges between 30% and 50%.

### 3.2. Rheological Analysis

Viscosity measurements were carried out using a 20 mL concentric cylinder system in which temperature was controlled at 25 °C by a Peltier device.

Rheological tests showed that viscosity increases with TG concentration. For a shear rate of less than 10 s^−1^, the viscosity of the 0.6% TG solutions is approximately 480 mPa.s, while the 0.8% TG solutions reach a viscosity of 700 mPa.s. Likewise, the solutions with a concentration of 1.0% TG presented viscosities of 1500 mPa.s, significantly higher than those obtained for the other TG concentrations.

As the shear rate increases, the viscosity decreases to 30 mPa.s for 0.6% TG, 45 mPa.s for 0.8% TG, and 70 mPa.s for 1.0% TG. This is because higher concentrations of TG generate a stiffer molecular chain network via hydrogen bridges [10]. Moreover, the viscosity reduction with increasing shear rate is typical of pseudoplastic solutions [1], where macromolecular chains become disentangled and aligned in the direction of flow, decreasing the interactions between them [16]. This behavior was corroborated with the calculated parameters of the Herschel–Bulkley model (Table 1).

**Figure 1:**
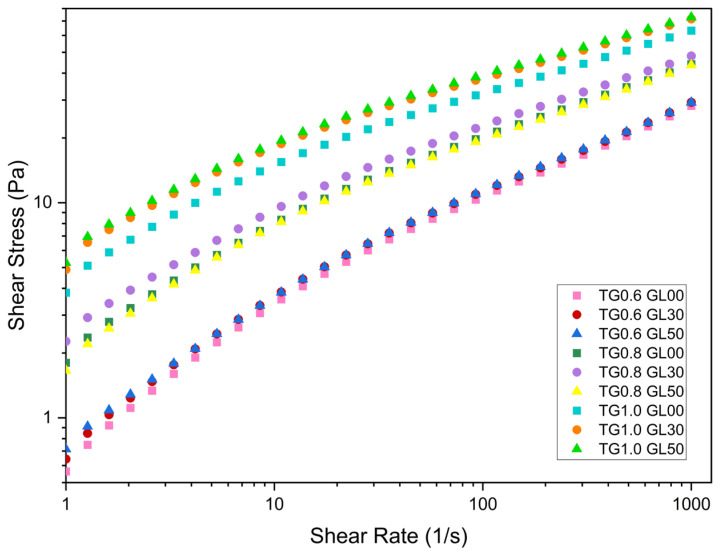
Pseudoplastic behavior of the flow curves for Tara gum solutions and glycerol mixtures.

GL possessing three hydroxyl groups (OH-) facilitates the formation of hydrogen bridges with the side chains of TG, which strengthens the network entanglement and increases the viscosity of the film-forming solutions [17,18]. However, as observed in Figure 1, varying GL content between 30% and 50% has a smaller impact on the flow curves than modifying TG concentration.

From Figure 1, it can be seen for all cases, the yield stress limit is affected by the concentration of GL and TG.

In Table 1, we can see that when OP was added to the mixtures, the flow coefficient (k) subtly decreased, while the flow behavior index (n) slightly increased, maintaining a shear-thinning behavior, with TG as the dominant component of the solution. Similarly, OP was observed to increase the values of the flow behavior index (n), indicating that its incorporation enhances the viscous behavior of the solution. However, since it does not exceed the threshold of 1, the fluid remains pseudoplastic.

On the other hand, the data indicate that increasing the concentration of TG (from 0.8% to 1.0% *w*/*v*) led to a decrease in the 12.7% value from 0.487 (Composition 1) to 0.428 (Composition 7), which is consistent with the previous findings reported by Wu et al. [1] and Santos et al. [19], who studied the rheology of TG at different concentrations. According to these authors, the pseudoplastic nature of TG solutions becomes more pronounced as their concentration increases. This suggests that, although OP contributes to a slight increase, TG remains the predominant factor in determining the rheological behavior, reinforcing its pseudoplastic tendency at higher concentrations.

Before the frequency sweeps, amplitude sweeps were performed from the lowest TG concentration (0.6%) to the highest (1.0%), selecting the shear deformation value within the linear region of 5%. Frequency sweeps were then carried out with a shear deformation of 5% over a frequency range of 1 to 100 rad/s. In the first stage, TG concentrations of 0.6%, 0.8%, and 1.0% were evaluated with different GL concentrations. In the second stage, mixtures of 0.8% and 1.0% TG, 30% and 50% GL, and varying OP concentrations were tested.

**Figure 2:**
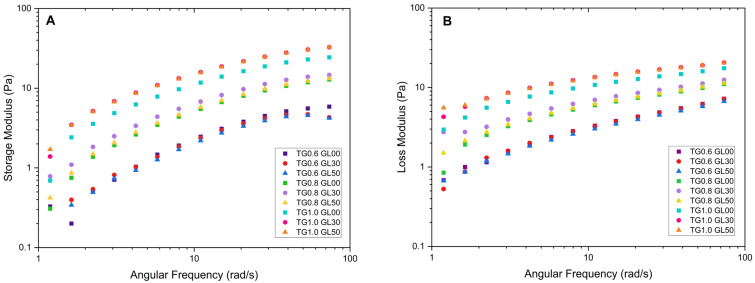
Effect of Tara gum (TG) and glycerol concentrations on the viscoelastic behavior of film-forming solutions: storage modulus (**A**) and loss modulus (**B**).

The storage modulus for the solutions with 1.0% TG shows a steep initial curve, followed by a decrease toward values close to zero. The highest peak of 62.10 Pa was recorded for the solution with 1% TG and 30% GL. Furthermore, it was observed that the storage modulus increases with angular frequency, which is attributed to the rearrangement of molecular chains due to the increase in frequency [17], as evidenced in Figure 2.

The loss modulus also increases with increasing angular frequency; however, it does not exceed the values reached by the storage modulus. The maximum value recorded was 20.454 Pa for the solution with 1.0% TG and 50% GL. In addition, the solutions with a higher concentration of TG exhibit moderate growth compared to the 0.6% solutions, which show a significant linear increment. This is due to an enlargement in the energy released, which causes the viscous component of the solution to predominate over the elastic component.

**Figure 3:**
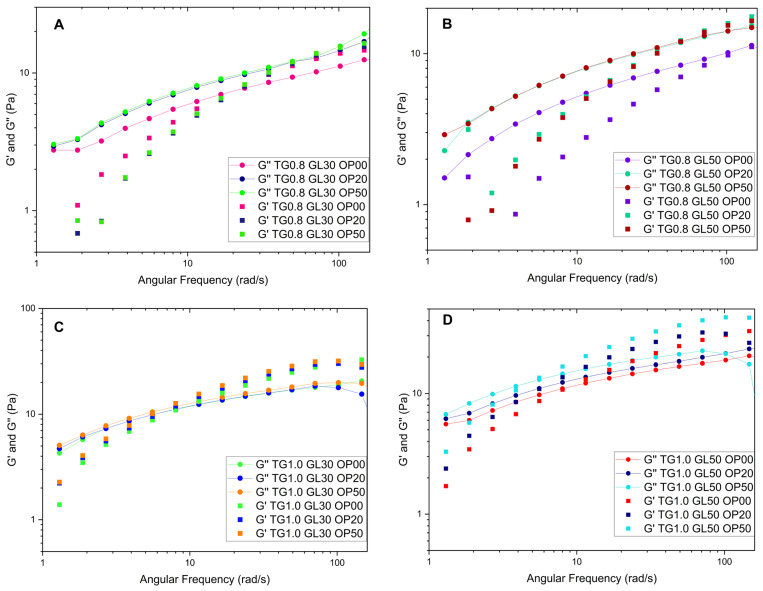
Influence of orange peel on viscoelastic properties, storage modulus (G′) and loss modulus (G″): (**A**) Compositions 1, 2, and 3; (**B**) Compositions 4, 5, and 6; (**C**) Compositions 7, 8, and 9; (**D**) Compositions 10, 11, and 12.

Figure 3 shows the behavior of some solutions. When OP is added to the solutions containing 0.8% TG, they behave as a liquid until a crossover point appears at 3.8 rad/s, after which they acquire a gelatinous character. The solutions with 1.0% TG observe a less-liquid behavior, since they comprise more TG and have higher values of the storage modulus at high frequencies, due to the increased crosslinking, forming a more cohesive network that can store more energy.

The addition of orange peel does not significantly affect the rheological behavior in terms of loss and storage modulus, with TG being the dominant component. At low frequencies, the loss modulus is higher, whereas at higher frequencies, the storage modulus prevails. This behavior has been observed in previous studies [1,10], where TG solutions exhibit an entangled solution behavior at higher frequencies.

A correlation of the frequency sweeps was performed using Maxwell’s equations, finding that the four-element model (*n* = 4) is adequate to accurately describe the behavior of the solutions. The values of λ_i_ and G_i_ are reported in Appendix A (Table A1).

Table 2 shows the storage and loss modulus adjustments using Maxwell’s equations. Solutions with 0.8 TG in their composition present a better interaction between TG, GL, and OP, increasing the storage modulus with increasing OP concentration. Solutions that are composed of 1.0% TG and high OP content show a smaller storage modulus by about 35%. This effect can be attributed, on the one hand, to the saturation of the polymer matrix due to the formation of intermolecular bonds between OP cellulose, TG, and GL [20,21]. On the other hand, it could also be related to the intrinsic nature of OP as a filler material [4], since an excess of its incorporation interferes with the cohesion of the polymeric network, affecting its structural integrity.

**Figure 4:**
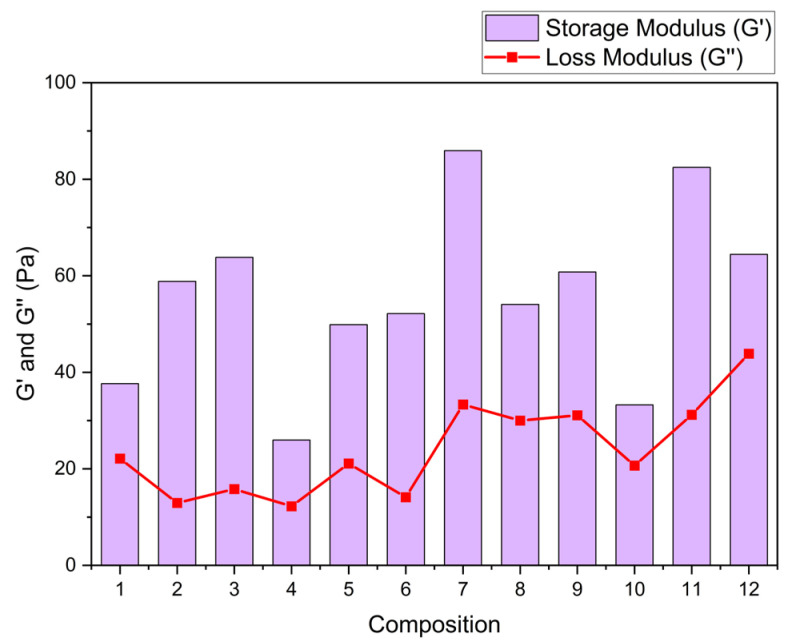
Importance of storage in respect to the loss modulus for the 12 compositions.

Figure 4 shows that the storage modulus G′, which reflects the stored elastic energy and elasticity of the matrix [6], increases markedly in the compositions with higher contents of TG (1.0%). This increment suggests a more organized and cohesive polymeric network, attributed to hydrogen-bonding interactions between the hydroxyl groups of the TG, the OP compounds (cellulose and pectin), and the GL. On the other hand, the loss modulus G″, related to the energy dissipated as heat and the viscosity of the material [10], shows a slight increment in the formulations with higher GL content (50%), indicating a higher flow capacity and higher plasticity.

It is important to emphasize that in this study, all rheological measurements were conducted at a controlled temperature of 25 ± 2 °C. Although the effect of temperature on the rheological properties was not investigated, this factor could have a significant influence on polymeric systems such as those evaluated.

### 3.3. Characterization of Glycerol, Tara Gum, and Orange Peel Films

#### 3.3.1. Thickness of the Films

The thickness values were within the range of 0.08–0.23 mm and were directly related to the solid concentration (TG and OP) in the film-forming solutions.

#### 3.3.2. Water Solubility, Water Vapor Permeability, and Contact Angle

As shown in Table 3, after 24 h of immersion, films without OP lost their structural integrity and exhibited significantly higher solubility compared to OP-containing formulations. Films incorporating OP retained their structural stability and exhibited a solubility reduction of up to 62.43%, indicating that OP enhances the water resistance of films. This effect can be attributed to the presence of cellulose, characterized by a highly ordered structure and lower hydrophilicity, that functions as a barrier restricting water penetration and consequently reducing solubility [22].

Viscoelastic properties, particularly the storage modulus, are directly associated with the cohesive strength of the polymer matrix [23], influencing both water resistance and mechanical durability. This behavior is evident in formulations reinforced with less hydrophilic components, such as cellulose [24] and chitosan [12]. As shown in Table 4, and in line with the solubility results, water vapor permeability varied as a function of TG and OP concentrations. Films with the lowest concentration of OP and 1.0% TG (specifically Compositions 8, 9, and 12) showed the lowest permeability values. In contrast, films with a higher concentration of OP and 0.8% TG exhibited the highest permeability values, reaching up to 0.0793 g mm/hm^2^ kPa in Composition 5.

The literature suggests that the permeability of hydrocolloid-based films increases with the hydrophilicity of the polymer [12]. In this case, the interaction between the three components affects permeability, as it decreases with higher concentrations of TG and OP. This is due to the formation of a more cohesive and effective barrier against vapor diffusion, probably due to the more compact structure of the polymer matrix, which reduces water vapor diffusion through the films [25]. Pectin and cellulose present in OP have antioxidant activity and reactive oxygen species, contributing to the improved barrier properties of the films [5,12,25]. On the other hand, a higher GL concentration resulted in a more hydrophilic polymeric matrix, causing a slight increase in permeability; a similar behavior was reported by Antoniou et al. [12] and Xue et al. [26]. However, the effect of TG and OP predominated in the permeability of the films.

**Figure 5:**
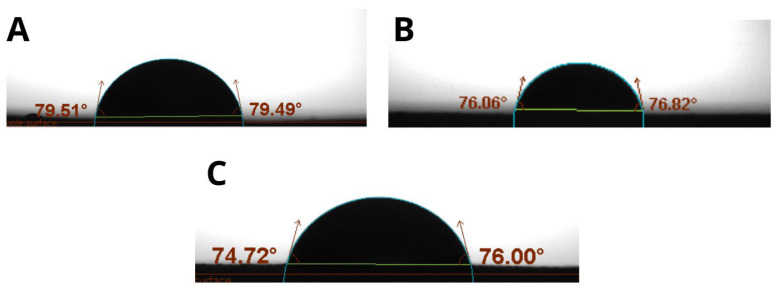
Maximum observed contact angles: (**A**) Composition 4: TG0.8 GL50 OP00; (**B**) Composition 9: TG1.0 GL30 OP50; (**C**) Composition 11: TG 1.0 GL50 OP20.

Water resistance is a crucial property for films applied as coatings [27]. The contact angle method is a reliable way to evaluate the degree of hydrophobicity. Films with a contact angle less than 65° are considered hydrophilic, while those with an angle greater than 65° are hydrophobic [27,28,29]. As shown in Figure 5, the results indicate intermediate behavior, with contact angles between 65° and 90°, suggesting greater liquid adhesion to the film than cohesion, congruent with hydrocolloid films [30]. The surface roughness increases with OP content, with the observation that the contact angles on the front side are up to 16.39% higher than on the reverse side, indicating increased hydrophobicity on that side due to its roughness.

This increase in surface roughness is closely related to the viscoelastic properties of the forming solutions. A higher storage modulus G′ suggests a more cohesive and rigid matrix [23], which can generate a rougher surface by increasing the contact angle due to the internal stress generated along the drying process [31,32]. Thus, the rheological properties are directly related to the surface structure and the hydrophobic behavior of the films.

#### 3.3.3. Fourier Transform Infrared Spectroscopy

It is necessary to note that the OP used was subject to an essential oil steam distillation process. This distillation process eliminates volatile compounds and essential oils, which leads to the reduction or elimination of the bands associated with terpenes and other volatile compounds present in the essential oil.

**Figure 6:**
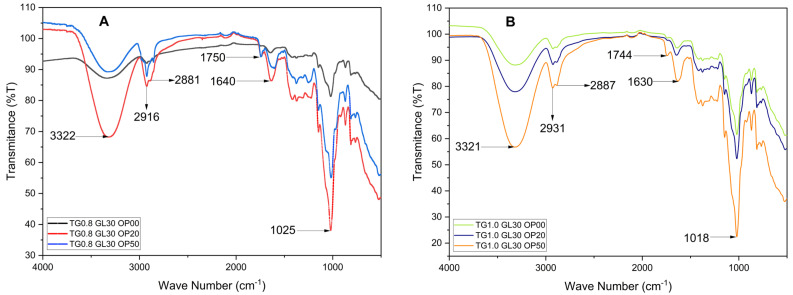
Effect of orange peel powder content on the FTIR spectra of Tara-gum-based films at 0.8% (**A**) and 1.0% (**B**) TG concentrations.

In Figure 6, a broad band is evident between 3600 and 3200 cm⁻^1^, corresponding to O–H stretching vibrations. The intensity and position of this band vary with OP concentration, suggesting hydrogen bonding between the hydroxyl groups of glycerol (GL) and Tara gum (TG), and the cellulose and pectin present in the orange peel [33]. In the 3000–2850 cm⁻^1^ region, C–H stretching vibrations typical of aliphatic chains are observed [17]. The peak near 1750 cm⁻^1^ corresponds to C=O stretching of carbonyl groups, likely from organic acids in the orange peel [22]. The band between 1600 and 1500 cm⁻^1^ may indicate aromatic structures, while those between 1300 and 1000 cm⁻^1^ reflect C–O ether bonds from OP [22] and C–OH groups of GL, as well as C–O–C glycosidic linkages from TG [10]. A notable shift in this region was detected, with the band moving from 1014–1033 cm⁻^1^ (in pure components) to 1018–1025 cm⁻^1^ in the blended films, depending on TG and OP content. This shift likely arises from changes in the local chemical environment of C–O-related groups due to intermolecular interactions such as hydrogen bonding. Although primarily manifested near 3300 cm⁻^1^, such interactions can influence other vibrational modes indirectly. Finally, the region below 1000 cm⁻^1^ represents the fingerprint zone, where the films show variations in transmittance values linked to their unique structural features.

TG is mainly composed of mannose and galactose, polysaccharides rich in hydroxyl groups (-OH), which are highly polar and due to their hydrophilic character have a strong affinity to form hydrogen bonds [18,34]. These interactions occur especially with the flavonoids, cellulose, and hemicelluloses present in OP, generating a more cohesive network that influences the mechanical strength, permeability, and solubility of the films [4,22]. The GL structure facilitates the additional formation of hydrogen bonds with TG (Figure 5); the intensity of the OH band is directly proportional to GL concentration, which will increase the solubility, flexibility, and elongation of the films. In addition, the carboxyl and hydroxyl groups of the OP compounds interact with the GL, promoting a more homogeneous distribution of its components within the polymeric matrix. This effect stabilizes the polymeric network and improves film uniformity, although it also affects hydrophobicity by increasing the surface polarity [35].

### 3.4. Tensile Strength (TS)

The initial tensile strength of the films was 13.93 ± 0.44 MPa for the 0.8% TG concentration and 19.41 ± 0.67 MPa for 1.0% TG, reaching a maximum of 27.93 MPa with 50% OP in the TG 0.8 G 30 formulation. It was evidenced that the combination of TG and OP strengthened the polymeric matrix, improving the mechanical resistance by up to 50.21%. The increase in GL reduced the tensile strength due to the decrease in the intermolecular forces in the polymeric chains [36,37]. Tensile strength presented values between 10 and 20 MPa, being proportional to OP concentration. Previous studies containing nanocellulose, polyvinyl alcohol, and glutaraldehyde showed similar results [21,38]. Typically, food films report tensile strength values between 15 and 25 MPa, ensuring durability and flexibility, which is consistent with the formulations of TG between 0.8% and 1.0% with 50% OP.

#### 3.4.1. Elongation (%)

The tests reported low elongation percentages: 2.86% for Composition 9 and 6.27% for Composition 8, both with 1.0% TG, suggesting a more compact polymeric matrix. In contrast, compositions with 0.8% TG and 50% GL showed higher elongations, 31.07% (Composition 5) and 40.68% (Composition 4), indicating that OP concentration reduces elongation due to the structural nature of cellulose [22]. GL acted as a plasticizer, increasing flexibility by 78.86%. A higher GL concentration increases elongation by weakening intermolecular forces and favoring the formation of hydrogen bonds, which improves flexibility but decreases tensile strength [39,40].

**Figure 7:**
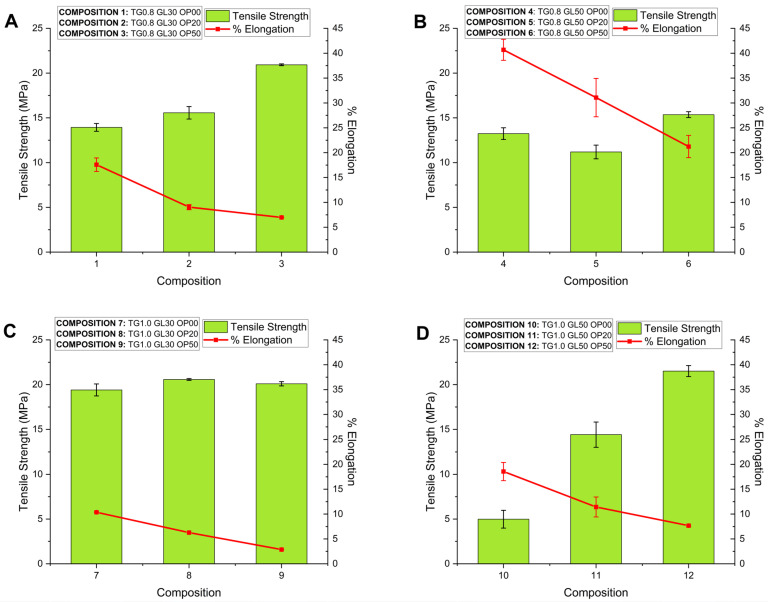
Mechanical performance of biopolymer films: effect of film composition on tensile strength and elongation for compositions with varying orange peel content (0%, 20%, 50%): (**A**) TG0.8 GL30; (**B**) TG0.8 GL50; (**C**) TG1.0 GL30; (**D**) TG1.0 GL50.

Analyzing Figure 7A,B,D, the important effect of OP on the tensile strength as well as elongation of the films can be observed. That effect is diminished in the compositions presented in Figure 7C. The impact of TG on the mechanical properties can be appreciated by comparing Figure 7B,D. The combination of high OP and TG concentrations produces an important increase in tensile strength and at the same time a reduction in the elongation of the films. The results suggest that important interactions take place between the film components.

Additionally, film thickness exhibited a direct influence on mechanical properties. Formulations with a higher solid content, such as those containing 1.0% TG and 50% OP, produced thicker films associated with higher TS (20.57 MPa) and YM values (402.34 MPa), suggesting a denser and more rigid polymer matrix. In contrast, thinner films exhibited greater elongation, reflecting a more flexible structure. These findings indicate that thickness, governed by the composition of the film-forming solution, plays a modulatory role in the mechanical behavior of films.

#### 3.4.2. Young’s Modulus (YM)

Figure 7 shows that films with 1.0% TG have slightly higher Young’s modulus values compared to those containing 0.8%. The highest values were 400 MPa and 402 MPa for Compositions 3 and 9 (See Appendix C, Table A3), which also have the highest concentrations of OP. This increase in the Young’s modulus is due to the increased stiffness in the film, caused by the molecular interactions between OP and TG, which strengthen the polymeric matrix [4,41].

Food coatings showed a Young’s modulus between 10 and 500 MPa, with greater flexibility in those formulations with more plasticizer. Films with 0.8% TG are more flexible, which is suitable for conforming to irregular surfaces, while those containing 1.0% TG have a higher Young’s modulus, suggesting a higher stiffness, preferable for foods requiring a stronger barrier.

The YM exhibited a broad range from 27 to 402 MPa, indicating the significant influence of each component and their interactions. The important role of OP in increasing the YM can be observed (See Appendix C, Table A3), showing a direct proportionality to OP concentration. For instance, in formulations with 0.8% TG and 30% G, the YM increased from 79.54 MPa in the absence of OP to 400.41 MPa with 50% OP. A similar trend was observed in formulations with 1.0% TG, where the YM reached its highest value of 402.34 MPa.

TG also plays a key role in the YM. Comparing samples 1 and 7, which do not contain OP and have the same glycerol content (30%), the YM increased from 79.54 MPa to 186.99 MPa when TG increased from 0.8% to 1.0%. Furthermore, glycerol’s plasticizing effect is evident when comparing samples with 30% and 50% GL, as a higher GL content leads to a decrease in the YM, highlighting its impact on the mechanical rigidity of the films.

### 3.5. Mechanical-Dynamic Analysis

For the mechanical-dynamic analysis of the solutions and films, it is noted that the storage modulus was obtained by applying a tangential force to the surface of the solution, while the Young’s modulus was determined by applying a normal force to the film. Both moduli are linked to the elasticity and energy stored in the material. Film-forming solutions and biofilms, composed of polymeric chains, exhibit both viscous and elastic behavior, which we previously defined as viscoelastic behavior.

The relationship between the storage modulus (G′) and Young’s modulus (YM) is a key factor in linking the rheological properties of the film-forming solutions to the mechanical properties of the corresponding films. These properties, although measured in different contexts, are connected because they describe the response of the material to deformation, either in the fluid (rheology) or solid (mechanical) state.

G′ is a parameter that describes how the solution components interact to form a cohesive network. During the film-formation process, which involves evaporation of the solvent, these interactions determine the final structure of the polymer network, which directly influences the YM.

**Figure 8:**
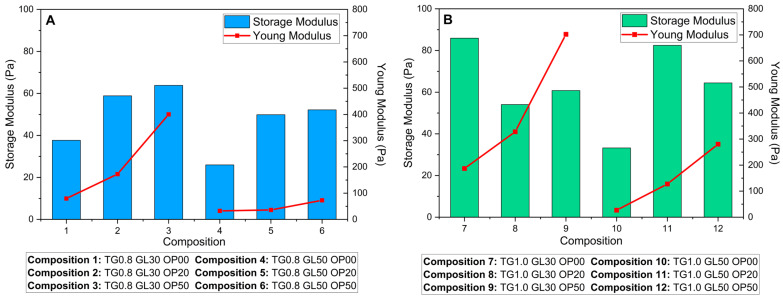
Comparison of the storage modulus (rheological) and Young’s modulus (mechanical): (**A**) films with 0.8% TG; (**B**) films with 1.0% TG.

Figure 8 compares the G′ of the liquid phase with the YM of the solid films, revealing differences based on TG concentration. In solutions with 0.8% TG (Figure 8A), a direct relationship between the G′ and YM is observed, where higher G′ values correspond to stiffer films. However, in solutions with 1.0% TG (Figure 8B), the relationship becomes more complex, suggesting a stronger synergistic effect among the components. This deviation could be attributed to more intense molecular interactions at higher TG concentrations, which under certain mixing conditions, lead to an increase in viscosity and a transition from a dilute to a concentrated regime [42,43]. In this context, the interactions between the components become more intricate, resulting in variations in the structural rigidity of the films.

The solutions exhibit pseudoplastic behavior, characterized by a decrease in viscosity under increasing shear stresses. This behavior suggests that the matrix possesses some elasticity, which favors the formation of more uniform and flexible films due to a homogeneous distribution of polymers. A high G′ in solutions with a higher TG concentration indicates a higher flow resistance and stronger viscoelastic behavior. This attribute predicts a higher stiffness in the final film, aligning with the increase in related mechanical properties, as we can see in Figure 8.

Data analysis was performed using IBM SPSS Statistics version 21. SPSS software was used to analyze the correlation between the two modules. First, normality tests were performed to determine whether parametric or nonparametric statistics should be applied, concluding that the data follow a normal distribution. Subsequently, a Pearson’s correlation index was calculated, which showed a significant relationship between the G′ and YM. This relationship is direct, with a positive Pearson’s coefficient of 0.840, indicating a high correlation between the two moduli.

Through a logarithmic multiple regression model, the relationship between the Young’s modulus (YM) and several predictor variables, including the storage modulus (G′) and the proportions of Tara gum (TG), glycerol (GL), and orange peel (OP), was evaluated. Calculations were performed with the logarithmic values of the YM and G′, obtaining the following equation:

Log(YM) = 6.813 + 0.669 Log(G′) + 0.467 (TG) **−** 0.024 (GL) + 0.002 (OP), (10)

In this equation, the coefficient associated with log(G′) is equal to 0.669, indicating that a 1% increase in the storage modulus generates an approximate 0.669% increase in log(YM), assuming all other variables are held constant. The coefficient of 0.467 associated with TG indicates that an increase in its concentration will cause a significant rise in the YM (*p* = 0.004). This can mean that TG, being a polysaccharide with hydroxyl groups, facilitates the interaction with other polymer chains like pectin and cellulose, which improves the mechanical strength of films [18,34]. Its positive effect on the mechanical properties of films suggests that increasing TG enhances its interaction with other matrix components through hydrogen bonding, a behavior that has also been observed in other studies on gums [42,44]. On the other hand, the proportions of GL and OP have a less significant impact. For each unit increase in the GL ratio, the Young’s modulus decreases slightly (−0.024), reflecting a softening effect of the plasticizer on the interactions between the polymer chains. In contrast, the proportion of OP has a positive effect (0.002), which strengthens the structural cohesion of the films.

The model as a whole presents an R^2^ = 0.928, indicating that 92.8% of the variation in log(YM) can be explained by the included predictor variables. In terms of synergy, the storage modulus exhibits a strong relationship with the Young’s modulus, while the specific interactions between the components (TG, OP, and GL) play a decisive role in the final material properties. In particular, an increase in TG concentration enhances these interactions, generating a synergistic effect that influences the structural and mechanical properties of the films.

Due to its strong explanatory power, this model could be applied in the design and development of other biopolymeric film systems, facilitating the engineering of materials with optimized mechanical properties. In this context, the present study not only demonstrates the interdependence between rheology and mechanics but also validates the feasibility of mathematically modeling these interactions, making a notable contribution to biopolymer materials science.

## 4. Conclusions

The analysis of the mechanical, rheological, and barrier properties revealed a complex relationship between the composition of Tara gum (TG), orange peel (OP), and glycerol (GL) films, highlighting a synergistic effect among these components. A strong correlation (r = 0.840) was observed between the storage modulus (G′) of the film-forming solutions and the Young’s modulus (YM) of the corresponding films, suggesting that more cohesive solutions yield stiffer films. However, this relationship is not strictly linear, as additional factors such as polymer–plasticizer interactions significantly influence the final material properties.

The synergy between Tara gum (TG) and orange peel (OP) plays a crucial role in optimizing the polymeric matrix. TG contributes to film stiffness and strength, while OP improves structural cohesion and stability. Glycerol (GL) acts as a plasticizer, increasing flexibility but lowering stiffness. This interaction reduced film solubility by up to 62.43%, improved mechanical strength by 50.21%, and increased flexibility by 78.86%. Additionally, the combination of TG and OP decreased water permeability, indicating a more compact matrix. FTIR confirmed a cohesive network formation, and contact angle measurements showed increased hydrophobicity with higher TG and OP content, suggesting enhanced moisture resistance. Rheological analysis revealed that a higher storage modulus (G′) corresponds to a more structured and mechanically stable matrix, influencing both mechanical and barrier properties.

The strong correlation between rheological and mechanical properties underscores the importance of understanding the combined effect of these biopolymers in the development of sustainable and high-performance coatings. This approach not only quantifies the interdependence between viscoelasticity and stiffness but also provides a novel analytical framework for the design and optimization of biopolymers. Given the statistical robustness and applicability of the model that correlates the elastic modulus to the Young’s modulus, it may be extended to other biopolymeric film systems, facilitating the development of sustainable and high-performance coatings for various industrial applications.

## Figures and Tables

**Table 1:** Herschel and Bulkley parameters for the different film-forming solutions.

#	Composition	k (Pa.s)	n	T_o_	R^2^
1	TG0.8 GL30 OP00	2.113 ± 0.036	0.487 ± 0.002	1.238 ± 0.028	0.9565 ± 0.0003
2	TG0.8 GL30 OP20	1.991 ± 0.010	0.580 ± 0.002	1.151 ± 0.013	0.9552 ± 0.0001
3	TG0.8 GL30 OP50	1.942 ± 0.010	0.583 ± 0.003	1.269 ± 0.010	0.9551 ± 0.0003
4	TG0.8 GL50 OP00	1.883 ± 0.013	0.487 ± 0.001	0.696 ± 0.005	0.9602 ± 0.0001
5	TG0.8 GL50 OP20	1.868 ± 0.005	0.584 ± 0.002	1.058 ± 0.003	0.9567 ± 0.0002
6	TG0.8 GL50 OP50	1.915 ± 0.013	0.579 ± 0.002	1.087 ± 0.003	0.9577 ± 0.0001
7	TG1.0 GL30 OP00	4.582 ± 0.015	0.428 ± 0.002	2.890 ± 0.008	0.9396 ± 0.0003
8	TG1.0 GL30 OP20	3.392 ± 0.006	0.545 ± 0.003	2.638 ± 0.008	0.9384 ± 0.0003
9	TG1.0 GL30 OP50	3.489 ± 0.008	0.545 ± 0.002	2.789 ± 0.012	0.9384 ± 0.0002
10	TG1.0 GL50 OP00	4.712 ± 0.012	0.426 ± 0.002	3.244 ± 0.011	0.9387 ± 0.0002
11	TG1.0 GL50 OP20	3.726 ± 0.010	0.539 ± 0.002	3.125 ± 0.005	0.9332 ± 0.0002
12	TG1.0 GL50 OP50	4.318 ± 0.010	0.529 ± 0.001	3.873 ± 0.010	0.9281 ± 0.0001

**Note:** Considering the concentrations of Tara gum (TG) (0.8–1%), glycerol (GL) (30–50%), and orange peel (OP) (0–50%). Values represent the mean ± standard deviation from three independent repetitions. Each repetition involved fitting the Herschel–Bulkley model to rheological data collected under identical conditions. The standard deviation of R^2^ reflects the variability of model fitting across repetitions.

**Table 2:** Calculated values of the storage modulus (G′) and loss modulus (G″).

#	Composition	Storage Modulus (Pa)	Loss Modulus (Pa)	%MSE_total_
1	TG0.8 GL30 OP00	37.6635 ± 0.2234	22.1298 ± 0.2419	1.0137
2	TG0.8 GL30 OP20	58.8527 ± 0.2587	12.9443 ± 0.2587	3.7771
3	TG0.8 GL30 OP50	63.8328 ± 0.2789	15.8221 ± 0.1330	4.1238
4	TG0.8 GL50 OP00	25.9653 ± 0.0902	12.2364 ± 0.0943	0.7428
5	TG0.8 GL50 OP20	49.8739 ± 0.2869	21.1021 ± 0.2269	1.2493
6	TG0.8 GL50 OP50	52.1687 ± 0.2121	14.1370 ± 0.2380	2.9916
7	TG1.0 GL30 OP00	85.9355 ± 0.2343	33.3463 ± 0.1114	0.5597
8	TG1.0 GL30 OP20	54.0570 ± 0.2000	30.0054 ± 0.1342	0.6281
9	TG1.0 GL30 OP50	60.7911 ± 0.1334	31.1088 ± 0.0906	0.4631
10	TG1.0 GL50 OP00	33.2621 ± 0.0958	20.6917 ± 0.0906	0.3044
11	TG1.0 GL50 OP20	82.4723 ± 0.2123	31.2105 ± 0.1280	1.7796
12	TG1.0 GL50 OP50	64.4366 ± 0.1000	43.8726 ± 0.1131	0.1561

**Note:** The tests were carried out at an angular frequency ranging from 1 to 120 rad/s. The total mean squared error (MSE) was calculated as the sum of the individual MSE values for G′ and G″, calculated as the total percentage error: %MSE = %MSEG′ + %MSEG″.

**Table 3:** Percentage solubility of films.

#	Composition	% Solubility	#	Composition	% Solubility
1	TG0.8 GL30 OP00	72.2336 ± 0.4632 ^a^	7	TG1.0 GL30 OP00	53.4893 ± 3.0943 ^b^
2	TG0.8 GL30 OP20	24.3941 ± 1.8814 ^cd^	8	TG1.0 GL30 OP20	49.2199 ± 3.2398 ^bc^
3	TG0.8 GL30 OP50	41.8904 ± 4.5794 ^bcd^	9	TG1.0 GL30 OP50	39.8970 ± 1.7636 ^bcd^
4	TG0.8 GL50 OP00	54.1691 ± 4.1751 ^b^	10	TG1.0 GL50 OP00	41.2269 ± 2.3216 ^bcd^
5	TG0.8 GL50 OP20	22.5797 ± 2.3995 ^cd^	11	TG1.0 GL50 OP20	45.6388 ± 0.6548 ^bc^
6	TG0.8 GL50 OP50	20.3508 ± 5.7969 ^d^	12	TG1.0 GL50 OP50	40.1387 ± 1.5464 ^bcd^

**Note:** Different letters indicate significant differences according to Tukey’s test (*p* < 0.05).

**Table 4:** Film permeability.

#	Composition	Permeability (gmm/hm^2^ kPa)	#	Composition	Permeability (gmm/hm^2^ kPa)
1	TG0.8 GL30 OP00	0.0528 ± 0.0031 ^bc^	7	TG1.0 GL30 OP00	0.0183 ± 0.0073 ^ab^
2	TG0.8 GL30 OP20	0.0645 ± 0.0067 ^c^	8	TG1.0 GL30 OP20	0.0158 ± 0.0064 ^ab^
3	TG0.8 GL30 OP50	0.0503 ± 0.0114 ^bc^	9	TG1.0 GL30 OP50	0.0151 ± 0.0051 ^a^
4	TG0.8 GL50 OP00	0.0639 ± 0.0051 ^c^	10	TG1.0 GL50 OP00	0.0165 ± 0.0010 ^ab^
5	TG0.8 GL50 OP20	0.0793 ± 0.0099 ^d^	11	TG1.0 GL50 OP20	0.0175 ± 0.0036 ^ab^
6	TG0.8 GL50 OP50	0.0745 ± 0.0103 ^d^	12	TG1.0 GL50 OP50	0.0131 ± 0.0005 ^a^

**Note:** Different letters indicate significant differences according to Tukey’s test (*p* < 0.05).

**Figure A1:**
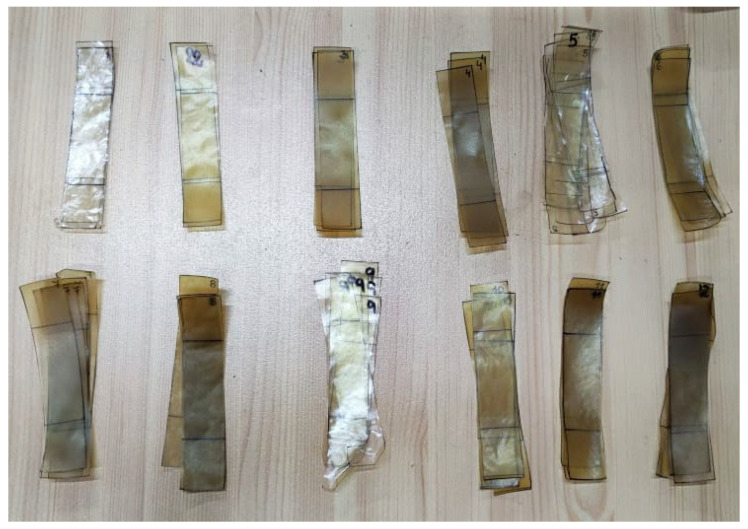
Final films.

## Data Availability

The data supporting the findings of this study are openly available in Mendeley Data at https://doi.org/10.17632/ztznrhmsvh.1 (Ortiz Cabrera, N. J.; Miranda, L., 2025).

## Appendix A

**Table A1:** Parameters λ_i_ and G_i_ from the Four-Element Maxwell Model.

#	Composition	G′ (Pa)				G″ (Pa)				λ_1_ (s)	λ_2_ (s)	λ_3_ (s)	λ_4_ (s)
		G_1_′	G_2_′	G_3_′	G_4_′	G_1_″	G_2_″	G_3_″	G_4_″				
1	TG0.8 GL30 OP00	36.7195	0.8921	0.0470	0.0046	18.2947	3.0912	0.7153	0.0284	0.0026	0.0171	0.071	0.4556
2	TG0.8 GL30 OP20	58.2956	0.4730	0.0750	0.0090	12.1746	0.7287	0.0362	0.0046	0.0027	0.0226	0.1024	0.4865
3	TG0.8 GL30 OP50	62.6390	0.6613	0.3665	0.1658	15.0197	0.7785	0.0219	0.0018	0.0064	0.0019	0.0805	0.4436
4	TG0.8 GL50 OP00	25.6624	0.2457	0.0472	0.0098	11.8080	0.3620	0.0638	0.0024	0.0125	0.0125	0.0618	0.3145
5	TG0.8 GL50 OP20	48.8098	0.9989	0.0572	0.0078	20.6085	0.4081	0.0854	0.0071	0.0033	0.0650	0.0135	0.4014
6	TG0.8 GL50 OP50	51.1893	0.8324	0.0939	0.0529	13.0736	0.9896	0.0683	0.0053	0.0027	0.0158	0.0893	0.5140
7	TG1.0 GL30 OP00	85.4421	0.4263	0.0637	0.0032	32.6352	0.6668	0.0426	0.0015	0.0017	0.007	0.0451	0.3206
8	TG1.0 GL30 OP20	53.0965	0.9432	0.0155	0.0016	29.2252	0.7263	0.0483	0.0053	0.009	0.009	0.0475	0.3505
9	TG1.0 GL30 OP50	60.2448	0.4946	0.0457	0.0058	30.6586	0.4090	0.0405	0.0005	0.0077	0.0434	0.4452	0.1517
10	TG1.0 GL50 OP00	31.8860	1.0003	0.37281	0.0029	12.9747	6.6379	0.1983	0.8805	0.0138	0.0138	0.0532	0.3594
11	TG1.0 GL50 OP20	81.8514	0.5868	0.0260	0.0078	30.1794	0.7283	0.3000	0.0026	0.0029	0.0117	0.0615	0.4253
12	TG1.0 GL50 OP50	64.0129	0.3839	0.0348	0.0048	43.0627	0.7227	0.0806	0.0065	0.0122	0.0276	0.0924	0.45805

The parameters G_i_ represent the elastic moduli associated with each relaxation time λ_i_. Values of G_i_′ correspond to the storage modulus (G′), while G_i_″ values correspond to the loss modulus (G″).

## Appendix B

**Table A2:** Abbreviations and symbols used in this study.

Symbol/Term	Description	Unit
TG	Tara gum	%
GL	Glycerol	%
OP	Orange peel	%
n	Behavior index	-
k	Flow coefficient	-
G′	Storage modulus	Pa
G″	Loss modulus	Pa
%S	Water solubility	%
WVP	Water vapor permeability	gmm/hm^2^ kPa
WVTR	Water vapor transmission rate	g/m^2^ h
CA	Contact angle	°
TS	Tensile strength	MPa
%E	Percent elongation	%
YM	Young’s modulus	MPa
λ_i_	Relaxation time	s

## Appendix C

**Table A3:** Mechanical test values.

#	Composition	Tensile Strength (MPa)	Elongation (%)	Young’s Modulus (MPa)
1	TG0.8 GL30 OP00	13.9333 ± 0.4360	17.5863 ± 1.3548	79.5368 ± 6.5603
2	TG0.8 GL30 OP20	15.5687 ± 0.6984	9.0451 ± 0.5165	172.7479 ± 17.0106
3	TG0.8 GL30 OP50	20.9288 ± 0.1032	6.9755 ± 0.0605	400.4113 ± 4.5974
4	TG0.8 GL50 OP00	13.2381 ± 0.6547	40.6834 ± 2.1129	32.5953 ± 2.3027
5	TG0.8 GL50 OP20	11.1897 ± 7693	31.0786 ± 3.8572	36.1840 ± 2.3840
6	TG0.8 GL50 OP50	15.3595 ± 0.3307	21.2313 ± 2.2351	72.7596 ± 5.9332
7	TG1.0 GL30 OP00	19.4088 ± 0.6725	10.3780 ± 0.0813	186.9949 ± 5.1634
8	TG1.0 GL30 OP20	20.5732 ± 0.0964	6.2704 ± 0.0186	328.1069 ± 2.4958
9	TG1.0 GL30 OP50	20.0974 ± 0.2368	2.8614 ± 0.0180	402.3428 ± 3.9263
10	TG1.0 GL50 OP00	4.9825 ± 0.9942	18.5617 ± 1.8269	27.0286 ± 6.1377
11	TG1.0 GL50 OP20	14.4170 ± 1.3978	11.4221 ± 2.0106	127.4136 ± 11.1279
12	TG1.0 GL50 OP50	21.5162 ± 0.6162	7.6766 ± 0.1426	280.4390 ± 12.9315
